# Good long-term outcome of the untreated contralateral hip in unilateral slipped capital femoral epiphysis: Forty hips with a mean follow-up of 41 years

**DOI:** 10.1007/s11832-014-0611-2

**Published:** 2014-09-30

**Authors:** Anders Wensaas, Ragnhild B. Gunderson, Svein Svenningsen, Terje Terjesen

**Affiliations:** 1 Department of Orthopaedics, Akershus University Hospital, 1478 Lørenskog, Norway; 2 Department of Radiology, Oslo University Hospital, Rikshospitalet, Postboks 4950, Nydalen, 0424 Oslo, Norway; 3 Department of Orthopaedics, Sorlandet Hospital, Postboks 783 Stoa, 4809 Arendal, Norway; 4 Department of Orthopaedics, Oslo University Hospital, Rikshospitalet, Postboks 4950 Nydalen, 0424 Oslo, Norway

**Keywords:** Paediatric, Hip, Slipped capital femoral epiphysis, Contralateral, Prophylactic fixation, *α*-Angle

## Abstract

**Purpose:**

There is no consensus regarding prophylactic fixation of the contralateral hip in slipped capital femoral epiphysis (SCFE). In order to further study this question, we evaluated the long-term natural history of untreated contralateral hips.

**Methods:**

Forty patients treated for unilateral SCFE without evidence of subsequent contralateral slip during adolescence were reviewed with a mean follow-up of 36 years (range 21–50 years). The deformity after SCFE may demonstrate radiographic signs of cam-type femoroacetabular impingement. We, therefore, measured *α*-angles in the contralateral hips on anteroposterior (AP) and frog-leg lateral radiographs. The angles were compared with those of a control group of adults without SCFE. Five years after the radiographic examination, with a mean follow-up of 41 years, all patients were evaluated by telephone interview. As range of motion and deformity could not be examined, a modified Harris hip score (HHS) (maximum score of 91 points) was used. A modified HHS <76 points and/or radiographic osteoarthritis (OA) was classified as a poor long-term outcome.

**Results:**

The mean value of the AP *α*-angle was significantly higher in the contralateral hips in SCFE patients than in the control group (55° vs. 46°), while the mean value of the lateral *α*-angle was not. Abnormally high values for one or both *α*-angles were found in 16 contralateral hips (40 %), of which five patients had abnormal values for both *α*-angles and were considered to have had an asymptomatic contralateral slip. Five patients (13 %) had a poor outcome in the contralateral hip, of which three patients (8 %) had OA. There was a significant association between hips with both *α*-angles that were abnormal and poor outcome.

**Conclusions:**

Since the natural history showed good long-term radiographic and clinical outcome in 35 of 40 patients and only three had OA, we conclude that routine prophylactic fixation of the contralateral hip is not indicated.

## Introduction

No consensus has been reached regarding prophylactic fixation of the contralateral hip in unilateral slipped capital femoral epiphysis (SCFE), and there is no reliable method of predicting contralateral slip. The frequency of bilateral slip varies markedly from approximately 15 % to 60 % according to the literature [1–9]. The highest incidence has been reported by Billing and Severin [10], who used a sophisticated radiographic technique and found a frequency of bilateral slips of 80 %.

Before recommending prophylactic fixation, the long-term natural history of the contralateral hip without fixation should be established and compared with the outcome after prophylactic fixation. If the outcome was significantly better in hips with prophylactic fixation, this treatment could be recommended.

Mild asymptomatic contralateral slip could be revealed through a radiographic follow-up, since studies during the last several decades have shown that the deformity after SCFE can cause cam-type femoroacetabular impingement (FAI) [11–14]. No previous follow-up study has evaluated the untreated contralateral hip for radiographic signs of FAI. The long-term outcome of hips with FAI indicating possible ‘silent’ slips without treatment would be interesting in the discussion of prophylactic fixation.

The purposes of this study were to answer the following questions:

1. What is the frequency of radiographic signs of FAI indicating possible previous asymptomatic slip in the contralateral hip in patients with unilateral SCFE?
2. What is the long-term natural history of the contralateral hips with signs of FAI and in hips without such signs?

## Methods

This study was based on a previous follow-up study of patients treated for SCFE [8]. The patients were identified by searching through the radiographic archive of our hospital during the year 2006. We included patients that were living in the South-East area of Norway, with all radiographs available and a minimum follow-up of 20 years follow-up. Seventy-nine patients met the inclusion criteria. However, 15 patients were not included for the following reasons: nine patients had died during the follow-up time, five patients did not respond to the enquiry and one patient did not want to participate in the study. Sixteen patients (including two with bilateral slip) did not appear for the follow-up examination and were evaluated by telephone interview. Thus, 48 patients were examined with follow-up radiographs. Eight of these patients had bilateral slip and were, therefore, not included in this study. Thus, 40 patients (18 men and 22 women) with unilateral SCFE were included. The mean age at diagnosis was 13.8 years among the male patients and 12.6 years among the female patients. Thirty-five patients had been treated with in situ fixation alone (23 with screw fixation and 12 with bone peg epiphysiodesis) and five patients with in situ fixation combined with corrective femoral osteotomy. None of the patients underwent prophylactic fixation of the contralateral hip.

As part of a long-term study of hips with previous SCFE, a radiographic follow-up was performed 36 years (range 21–50 years) after treatment [8]. At this time, we did not focus in detail on the contralateral hips and no clinical scoring of those hips was performed. However, 5 years after this follow-up examination, we decided to evaluate the contralateral hip more thoroughly. For this purpose, we used the radiographs taken at the original follow-up study, while pain and function were evaluated 5 years later by telephone interview, with a mean follow-up of 41 years (range 26–55 years). The mean patient age was 55 years (range 38–68 years).

**Fig. 1:**
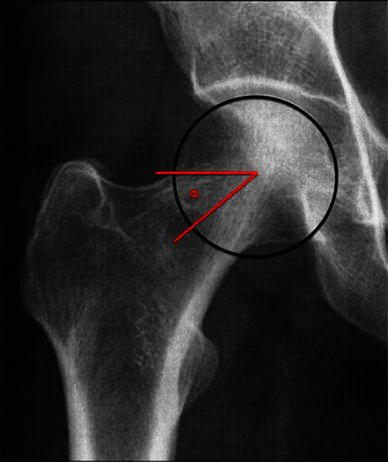
*α*-Angle in the anteroposterior (AP) view (*α*) in a normal hip

**Fig. 2:**
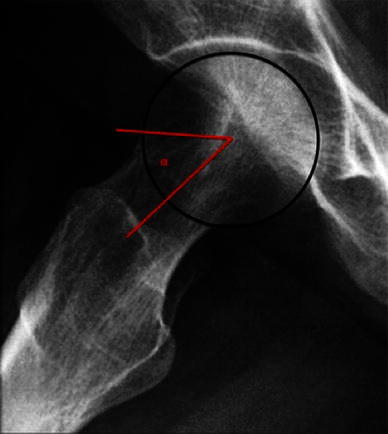
*α*-Angle in the frog-leg lateral view (*α*) in a normal hip

Post-slip deformity and FAI were evaluated with two radiographic measurements which have previously been shown to be abnormal in patients with SCFE compared to a control group [14]. The *α*-angle on the anteroposterior (AP) (Fig. 1) and frog-leg lateral radiographs (Fig. 2) were measured according to Gosvig et al. [15] and Nötzli et al. [16], respectively. An asymptomatic slip in the contralateral hip was considered to be present if both *α*-angles were abnormal. Osteoarthritis (OA) was defined according to Jacobsen and Sonne-Holm [17] as a minimum joint space width <2 mm in the upper weight-bearing part of the joint.

The control group consisted of 22 patients (10 male and 12 female) admitted for radiological examination of the hip(s) for lower limb symptoms but interpreted by the radiologists as normal. In bilateral cases, the right or left hip was randomly chosen. The mean age of the patients was 51 years (range 40–60 years). There were no significant differences in gender and age between the study population and the control group. The mean + 2 standard deviations (SDs) of the *α*-angle in the control group were used as limits of normal variation.

Hip function was measured with the Harris hip score (HHS) [18]. Since range of motion and deformity (9 points in the HHS) could not be evaluated in a telephone interview, we used a modified HHS where the maximum score was 91 points. A poor outcome was defined as a modified HHS <76 points (91 points minus 15 points) and/or radiographic signs of OA. This corresponds with the cut-off of 85 points (100 points minus 15 points) used in two previous studies [8, 14].

The study was approved by the Regional Ethical Committee and the Data Inspectorate, and all patients gave their informed consent prior to their inclusion in the study.

### Statistical analysis

SPSS version 18 (SPSS Inc., Chicago, IL) was used for the statistical analysis. Categorical data were analysed with the Pearson’s Chi-squared test and continuous data were analysed by the independent samples *t*-test (Student’s *t*-test). Differences were considered significant when the *p*-value was <0.05.

## Results

**Fig. 3:**
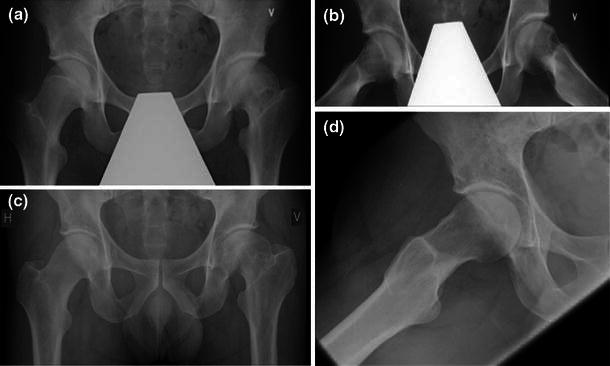
**a** AP and **b** frog-leg lateral radiographs at skeletal maturity (age 17 years) of a male patient treated for slipped capital femoral epiphysis (SCFE) of his left hip. **c** AP and **d** frog-leg lateral radiographs of the same patient at follow-up 32 years after diagnosis. Both *α*-angles in the right hip are abnormal (AP 74º and lateral 78º). There is no osteoarthritis and the modified Harris hip score (HHS) is 87 points

The contralateral hips in SCFE patients had a significantly higher AP *α*-angle than the control group (55° vs. 46°), while there was no significant difference for the lateral *α*-angle (Table 1). The *α*-angles were classified as normal or abnormal according to the threshold values estimated from the control group (mean + 2 SD). Fourteen contralateral hips had an abnormal *α*-angle on the AP radiographs and seven hips had an abnormal *α*-angle on the lateral radiographs. Both *α*-angles were abnormal in five hips and we considered that it was a high probability that they had had a previous asymptomatic slip (Fig. 3).

**Table 1:** Threshold values of normal variation [mean + 2 standard deviation (SD)] of the *α*-angles calculated from the measurements of 22 normal hips (control group) compared with the measurements in 40 contralateral hips in unilateral slipped capital femoral epiphysis (SCFE) at follow-up

Variable	Control group (*n* = 22)			Contralateral hips in SCFE (*n* = 40)		Difference between groups		
	Mean (range)	SD	Threshold value	Mean (range)	SD	Mean	95 % CI	*p*-Value^a^
*α*-Angle (AP)	46 (40–54)	3.8	54	55 (39–85)	12.6	9.1	4.8, 13.5	<0.001
*α*-Angle (lateral)	47 (36–70)	7.6	63	51 (30–82)	13.2	4.2	−1.1, 9.5	0.12

*n* number of hips, *SD* standard deviation, *CI* confidence interval, *AP* anteroposterior

^a^Independent samples *t*-test

Three patients (8 %) had OA in the contralateral hip at follow-up (Table 2). Of these, one patient had normal values for both *α*-angles and a modified HHS of 91 points, and one had abnormal *α*-angles and a modified HHS of 87 points. The third patient had normal *α*-angles and had undergone total hip replacement 42 years after diagnosis due to more pronounced pain; the HHS was not known preoperatively.

**Table 2:** *α*-Angles in the five patients with poor outcome [modified Harris hip score (HHS) <76 points and/or osteoarthritis (OA)] after SCFE

Patients	Radiographic measurements		Outcome	
	*α*-Angle (AP)	*α*-Angle (lateral)	Osteoarthritis	Modified HHS
1	39	61	+	91
2	85	78	+	87
3	51	55	+	THR
4	80	67	÷	67
5	43	30	÷	74

*Modified HHS* modified Harris hip score with a maximum score of 91 points (range of motion and deformity not evaluated), *AP* anteroposterior, *THR* total hip replacement

Thirty-eight of 40 patients had a good function (modified HHS >76 points) in the contralateral hip at follow-up. The mean modified HHS was 89 points (range 67–91 points). Twenty-eight patients had no symptoms at all from the contralateral hip, while nine patients had occasionally slight pain or discomfort without limping or functional limitations. Two patients had a modified HHS below 76 points, but none of them had OA (Table 2).

In total, five patients (13 %) had a poor outcome of the contralateral hip at follow-up. There were no significant associations between the presence of abnormal values for each of the *α*-angles and long-term outcome (Table 3). When both *α*-angles were abnormal, there was a significant association between these five hips and poor long-term outcome [odds ratio (OR) = 7.11, 95 % confidence interval (CI) 0.83–60.75, *p* = 0.047].

**Table 3:** Normal or abnormal *α*-angles related to outcome at follow-up (poor outcome: modified HHS <76 points and/or OA) in 40 contralateral hips in unilateral SCFE

Variable	Hips (*n*)	Outcome				
		Good	Poor	OR	95 % CI	*p*-Value^a^
*α*-Angle AP						
Normal	26	23	3	1.28	0.19, 8.72	0.80
Abnormal	14	12	2			
*α*-Angle lateral						
Normal	33	30	3	4.00	0.53, 30.28	0.16
Abnormal	7	5	2			
*α*-Angle combined						
Both normal	35	32	3	7.11	0.83, 60.75	0.047
Both abnormal	5	3	2			

*Modified HHS* modified Harris hip score with a maximum score of 91 points (range of motion and deformity not evaluated), *OR* odds ratio, *CI* confidence interval, *AP* anteroposterior

^a^Pearson’s Chi-squared test

## Discussion

In this series of 40 patients with unilateral SCFE, we found that 16 of 40 patients (40 %) had one or both *α*-angles above the upper normal limit, indicating contralateral FAI. This confirms the results of Fraitzl et al. [13] in a follow-up study of patients with unilateral SCFE and contralateral prophylactic fixation, where even the fixated contralateral hips had mean *α*-angles significantly higher than normal. We agree with them that this may be a result of a systemic disturbance of the development of the growth plate in affected individuals.

Recently, we reported that post-slip deformity expressed as abnormally high AP and lateral *α*-angles in previous SCFE hips were significantly associated with poor clinical and radiographic long-term outcome [14]. Abnormal *α*-angles in the contralateral hips are suggestive of previous asymptomatic epiphyseal slip. If one of the *α*-angles is abnormal, we think a previous ‘silent’ slip is possible. However, if both *α*-angles are abnormal, we consider that it is a high probability that a ‘silent’ slip has previously occurred.

Contralateral slips diagnosed at the initial admission or later during adolescence occur in 15–30 % of the patients [1, 2, 4, 8, 9, 19]. Asymptomatic slips detected by re-examination of previous radiographs or by post-slip deformity seen on follow-up radiographs increased the rate of bilaterality to 48–61 % [2, 3, 20]. Since different definitions of slip have been used, it is difficult to compare our results. If the five contralateral hips having both *α*-angles that are abnormally high are added to the eight patients treated for contralateral slip before skeletal maturity, the frequency of bilateral slip would be 27 %. There could be several reasons why our bilaterality rate is lower than those of the other studies. One possibility is that we underdiagnosed ‘silent’ slips, since we included only hips with both *α*-angles that were abnormal. On the other hand, it is possible that asymptomatic slips in the above-mentioned studies were overdiagnosed, since the limits of normal variation of the radiographic variables have not been definitely defined and reliability was not secured by inter-observer evaluation.

The long-term natural history of the contralateral hip in patients with SCFE is important in the discussion about prophylactic fixation. We found a good long-term prognosis, since OA occurred in only 3 of 40 contralateral untreated hips (8 %) in SCFE patients. A few other long-term studies exist, but comparison is difficult since different radiographic methods were used to define asymptomatic slip and to measure outcome. Hägglund et al. [2] evaluated 260 patients with a mean follow-up of 33 years and found 104 patients with asymptomatic contralateral slip diagnosed at the follow-up examination. They used the relationship between the centre of the femoral head and calcar femorale to define a previous slip and Ahlbäck’s classification of OA [21]. Twenty-eight of 104 (27 %) contralateral untreated ‘silent’ slips had OA at follow-up, but most were mild-grade OA. They recommended prophylactic pinning of the contralateral hip with the purpose of avoiding slipping and reducing the risk of OA. The opposite conclusion was drawn by Jerre et al. [20] based on a study of 61 patients treated for unilateral SCFE with a mean follow-up of 32 years. A contralateral asymptomatic slip was diagnosed at review of the radiographs in 14 patients. OA was recorded when the superior joint space was <3 mm. The frequency of OA was 31 % in hips with asymptomatic contralateral slip. Even though there was a higher rate of patients with OA in the group with non-treated slip, they did not recommend prophylactic fixation because this would have led to an unnecessary operation in more than half the patients. Regarding radiographic outcome, we defined OA as a minimum joint space width <2 mm in the upper weight-bearing part of the joint, which is a more strict definition of OA than that used in the long-term studies mentioned above [2, 20] and can be one explanation for the lower rate of OA in our study.

There are several limitations of the present study. First, it was retrospective with a limited number of patients. Second, the patients were not consecutive cases; thus, the rate of FAI and bilaterality could be less reliable. However, because our radiographic archive had been preserved since the 1950s, the great majority of radiographs of treated patients was still available and could be identified during our search. Thus, we consider the proportion of patients in the present study to be representative of all patients treated during the time period without any selection bias. Third, several individuals of the control group had symptoms and could hardly be considered representative of the general population; however, the radiologists described the radiographs as normal and the mean values of the radiographic measurements did not differ from those of earlier studies [15, 16, 22–24]. Fourth, a previous inter-observer study showed only moderate agreement in the measurements of the *α*-angle [14].

What were the consequences of not performing prophylactic contralateral fixation? From the original cohort of 43 patients with unilateral SCFE at primary admission, three patients had subsequent SCFE in the contralateral hip diagnosed and operated before skeletal maturity. The long-term outcome was good in two of these hips (slip angles of 24º and 38º, respectively), whereas OA occurred in the third (slip angle of 14º) patient, who underwent total hip replacement (THR) at the age of 50 years. Since OA developed in three contralateral hips evaluated in the present study, the total rate of contralateral OA was 4 of 43 hips (9 %). Although several patients had abnormal values in the *α*-angles used to define FAI, the natural history of the untreated contralateral hips showed good clinical and radiographic outcome in about 90 % of the hips.

A systematic review with a decision analysis [25] found that observation (rather than prophylactic fixation) was indicated when the probability of contralateral slipping after the time of primary diagnosis is less than 27 %. In another decision analysis [26], prophylactic contralateral fixation was recommended. However, a weakness of both these reviews is that no studies on the long-term outcome after contralateral prophylactic fixation exist. The longest follow-up study seems to be that of Fraitzl et al. [13], who had a mean follow-up of 14 years. They found a positive clinical impingement test in 5 of 16 hips, and several hips had increased *α*-angles, indicating that the prognosis is not necessarily good, even after prophylactic fixation. Moreover, a complication rate of more than 10 % has been reported after contralateral fixation [27, 28]. Although most were mild complications, serious complications like partial avascular necrosis and femoral fractures can occur [29].

Should contralateral prophylactic fixation be recommended based on the present results? Only if the long-term outcome is significantly better than that in hips with no routine fixation. So, the question is how much the OA rate would be reduced to after prophylactic contralateral fixation. First, it would not be reduced to 0 %, since the rate of OA in the general population is 2–3 % in adults under 60 years of age [30]. Second, it is questionable as to whether it would be reduced to a rate below the 9 % found in the present study, since previous studies have shown long-term OA in 9–19 % of contralateral hips without previous slips [2, 20]. We, therefore, conclude that routine prophylactic fixation is not indicated. However, prophylactic fixation seems rational in patients with increased risk of bilateral SCFE, such as children with endocrine disorders [31] and those who are particularly young [32]. In the remaining patients, a close follow-up with clinical and radiographic examinations every 6 months until skeletal maturity is important in order to treat contralateral slips as soon as possible.
